# Short-term changes in Bruch’s membrane opening-based morphometrics during the first week after trabeculectomy

**DOI:** 10.1007/s00417-022-05644-3

**Published:** 2022-04-08

**Authors:** Caroline Gietzelt, Jan-Niklas Lüke, Werner Adler, Thomas S. Dietlein, Alexandra Lappas, Verena Prokosch-Willing, Sigrid Roters, Ludwig M. Heindl, Claus Cursiefen, Philip Enders

**Affiliations:** 1 Department of Ophthalmology, Faculty of Medicine and University Hospital Cologne, University of Cologne, Kerpener Straße 62, 50924 Cologne, Germany; 2 Glaucoma Imaging Center University of Cologne (GICC), Faculty of Medicine and University Hospital Cologne, Kerpener Straße 62, 50924 Cologne, Germany; 3 Department of Medical Informatics, Biometry and Epidemiology, Friedrich-Alexander University Erlangen-Nuremberg, Erlangen, Germany

**Keywords:** Glaucoma, Optic nerve head, Trabeculectomy, Optic coherence tomography

## Abstract

**Purpose:**

To evaluate the dynamics of Bruch’s membrane opening-based morphometrics of the optic nerve head (ONH) using spectral-domain optical coherence tomography (SD-OCT) during the first week after glaucoma surgery by trabeculectomy with mitomycin C.

**Methods:**

Prospective, longitudinal analysis of 25 eyes of 25 patients treated by trabeculectomy. Twenty-four eyes had evaluable postoperative SD-OCT examinations. Bruch’s membrane opening minimum rim width (BMO-MRW) and peripapillary retinal nerve fiber layer (RNFL) thickness were analyzed at baseline before surgery, 1 day, 2 to 3 days, and 1 week after surgery. Changes compared to baseline were correlated to intraocular pressure (IOP).

**Results:**

One day after surgery, the mean BMO-MRW changed by + 26.17 µm, *p* = 0.001 (mean IOP reduction by 17.01 mmHg). This increase persisted on day 2–3 with a mean increase of BMO-MRW of + 25.33 µm, *p* = 0.001 (mean IOP reduction by 20.46 mmHg) and by week 1 with a mean BMO-MRW increase of + 33.17 µm, *p* < 0.001 (mean IOP reduction by 22.55 mmHg). The increase in BMO-MRW correlated significantly with the reduction of IOP on day 1 (Spearman’s rho *ρ* = 0.656, *p* = 0.003) and d2–3 (Spearman’s rho *ρ* = 0.479, *p* = 0.038). There was no statistically significant correlation found between the IOP and the increase in BMO-MRW in week 1.

RNFL thickness showed no significant changes at day 1 as well as days 2–3 (*p* ≥ 0.078, respectively). It showed a small but significant increase in week 1 by 3.94 µm, *p* = 0.015.

**Conclusions:**

Structural reversal of disc cupping in BMO-MRW occurs as early as 1 day after trabeculectomy and correlates to the extent of the IOP reduction. During the whole first week after surgery, a strong increase in BMO-MRW can be noted. The changes in BMO-based parameters need to be considered when evaluating patients’ longitudinal follow-up.


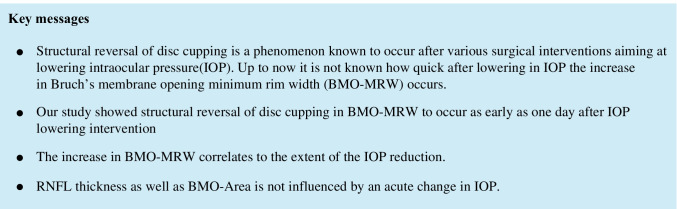


## Introduction

Optical coherence tomography (OCT) of the optic nerve head (ONH) has become an important diagnostic tool in the diagnosis and monitoring of glaucoma.

Two important parameters, which can be measured with SD-OCT of the ONH, are peripapillary retinal nerve fiber layer (RNFL) thickness and Bruch membrane opening minimum rim width (BMO-MRW). While the peripapillary RNFL thickness is measured in a circular scan at a 3.4 mm distance to the center of the BMO, the parameter BMO-MRW represents the minimum width of the neuroretinal tissue at the ends of the Bruch membrane opening [1]. Both parameters have shown superior diagnostic power to detect glaucoma compared to confocal laser scanning tomography (CSLT) in various clinical situations [2–8].

Knowledge about potential sources for bias is crucial for the correct interpretation of the imaging data. One such cause for bias is a phenomenon called structural reversal of disc cupping (SRDC). SRDC is known to occur after various surgical interventions aiming at lowering intraocular pressure (IOP) [9]. ONH changes related to SRDC have been described in fundoscopy as well as in confocal laser scanning tomography [9–13].

With the increased use of OCT in glaucoma diagnostics, several studies of our group could characterize SRDC to impact morphometric parameters of the ONH measured by spectral-domain OCT (SD-OCT). These were BMO-MRW and BMO-MRA.

A postoperative change resulting in an increase of BMO-based parameters was detected for different surgical strategies to treat glaucoma. After conventional trabeculectomy, BMO-MRW and -MRA were significantly increased up to more than 12 months postoperatively. After ab-interno trabeculectomy using electroablation of the trabecular meshwork in combination with cataract surgery as well as after glaucoma drainage device surgery, this effect was detectable for up to 6 months [14–16].

Up to now, the timing of the onset of SRDC still remains unclear. One study showed SRDC in cup-to-disc-ratio to occur as early as 24 h after IOP lowering intervention, however, with very small effect size and a sample size of 8 eyes of 6 patients [9]. Different other studies have shown SRDC in cup-to-disc-ratio as well as OCT parameters beginning 3 months after IOP lowering intervention [9, 14, 16]. In the previous retrospective studies by the Glaucoma Imaging Center of the University of Cologne (GICC), SRDC has been found 3 months postoperatively after IOP-lowering surgery. However, the timing of the onset could not be evaluated because the clinical standard or care does not included SD-OCT examinations of the ONH in the early postoperative follow-up. The objective of this prospective study was, therefore, to evaluate the onset and early dynamics of SRDC in SD-OCT parameters in the early postoperative phase after trabeculectomy.

## Methods

Patients hospitalized for trabeculectomy at our department were prospectively recruited and enrolled between October 2019 and June 2021. Overall, 591 patients underwent trabeculectomy at our center in this time period.

Due to several factors, among these the COVID-19 pandemic in Germany with suspension of active recruitment for all clinical studies, recruitment was performed in a limited number of weeks (*n* = 8) during the recruitment period. During these weeks, a total of 79 trabeculectomies were performed at our center. A total of 48% of these patients were not asked to participate due to limited availability of the examiners, for example on weekends. A total 10% did not meet inclusion and exclusion criteria, due to a lack of physical fitness, 9% decided not to participate in this study, and 33% (24 patients) could be included in the study. All patients provided written informed consent to participate. The study was approved by the local ethics committee of the University Hospital in Cologne (Approval no. 16–340) and was in accordance with the tenets of the Declaration of Helsinki.

Inclusion criteria were scheduled trabeculectomy of one eye at our center and the presence of a baseline SD-OCT examination of the ONH before surgery. Exclusion criteria were unsatisfactory image quality with a quality index of < 15 dB and manually not correctable segmentation errors. Follow-up SD-OCT examinations were conducted on day 1, day 2 or 3, and approximately week 1 (days 6–12) after surgery.

Patients’ medical history, including ophthalmologic diagnoses, best-corrected visual acuity (BCVA), topical medication at baseline, intraocular pressure (IOP) of both eyes at each examination, and epidemiologic data were collected from patients’ files. IOP was assessed by corneal rebound tonometry (Icare tonometer TA01i, Icare Finland Oy, Vantaa, Finland).

SD-OCT examinations of study and fellow were performed according to standard operating procedures of our center as described previously [14] using the Spectralis®-SD-OCT platform (Heidelberg Engineering GmbH, Heidelberg, Germany). Each examination consists of 24 scans with 48 points of measurement for BMO-MRW and three circular scans for RNFL. Only one SD-OCT examination was performed at each time point.

BMO-MRW and RNFL thickness were calculated by the software tool by Heidelberg Engineering, including a data export batch.

Surgery was conducted by a team of experienced glaucoma surgeons (TD, AL, SR, VPW, and PE). The surgical technique followed standard operating procedures for trabeculectomy of the center [14] with the application of mitomycin C.

Statistical analysis was performed using the software SPSS (version 25.0; IBM Corp, Armonk, New York, USA). We tested for normal distribution using Shapiro–Wilk Test, *p* > 0.05. The parameters BMO-MRW, RNFL thickness, as well as IOP at baseline, and all follow-up time points were normally distributed. (Shapiro–Wilk test for BMO-MRW at all time points *p* > 0.556, respectively; for RNFL thickness at all time points *p* > 0.531, respectively, for IOP at all time points *p* > 0.236 respectively, for BCVA *p* = 0.445). We used mixed regression analysis to check for differences between time points and Spearman’s *ρ* to analyze the correlation between IOP and morphometric parameters. The threshold for statistical significance was set to *p* < 0.05.

Follow-up examinations took place at three time intervals after surgery as described above. Not all patients had available examinations at all three time points due to clinical reasons (e.g., bed rest), patients’ wishes, or poor image quality (e.g., hyphema). Therefore, our analysis contains three subsets of baseline data with available FU examinations at day 1 (d1), day 2 to 3, (d2–3) and week 1 (w1). These subsets have different mean BMO-MRW and mean RNFL thickness values at baseline due to the divergence of eyes included in each subset. This approach was consistent with previous publications of our group [14, 15].

## Results

Twenty-four patients could be included in the study. One patient was excluded due to missing data due to withdrawal of informed consent on the first postoperative day due to vertigo. All patients had evaluable SD-OCT imaging data of the ONH to assess the neuroretinal tissue at least on one follow-up examination. On day 1 after surgery, evaluable SD-OCT was available in 18 patients, in day 23 in 20 patients and in week 1 in 14 patients.

One patient received a trabeculectomy of his fellow eye in the study period, therefore his fellow eye was excluded from the analysis of fellow eyes. All other fellow eyes were not treated by any surgical intervention during the study period (*n* = 23). Eleven patients enrolled in this study had primary open-angle glaucoma (POAG), eight patients had pseudoexfoliation glaucoma, one had pigment dispersion glaucoma, two had juvenile glaucoma (one because of Axenfeld Rieger’s anomaly, and the other patient because of idiopathic angle-dysgenesis), and two had secondary uveitic glaucoma. No study eye, but one fellow eye had previously received trabeculectomy, eleven study eyes and nine fellow eyes were pseudophakic.

Table 1 summarizes epidemiologic data.

**Table 1:** Epidemiologic data of study population

	Study eyes (*N* = 25)	Fellow eyes (*N* = 24)
Sex, *n* (%)		
Male	16 (67)	15 (65)
Female	8 (33)	8 (35)
Age (years)		
Mean ± SD	64.1 ± 14.0	63.9 ± 14.3
Range	32 to 89	32 to 89
Glaucoma diagnosis		
Primary open-angle glaucoma	11 (45.8)	
Pseudoexfoliation glaucoma	8 (33.3)	
Pigment dispersion glaucoma	1 (4.2)	
Juvenile glaucoma	2 (8.3)	
Secondary glaucoma (inflammatory, rubeosis, ICE syndrome, aphakia)	2 (8.3)	
Eye, *n* (%)		
Left	15 (62.5)	9 (39.1)
Right	9 (37.5)	14 (60.9)
Lens status at baseline, *n* (%)		
Phakic	11 (47.8)	14 (60.9)
Pseudophakic	12 (52.2)	9 (39.1)
Aphacic	0 (0)	0
BCVA at baseline (logMAR)		
Mean ± SD	0.17 ± 0.23	0.09 ± 0.14
Range	1.0 to − 0.1	0.4 to − 0.1
Spherical equivalent at baseline (dpt)		
Mean ± SD	− 0.60 ± 2.15	− 0.34 ± 1.93
Range	− 3.25 to + 6.75	− 4.0 to + 5.75
Intraocular pressure (IOP) at baseline (mmHg)		
Mean	30.04	17.39
Range	11 to 53	8 to 27
IOP at day 1 (mmHg)		
Mean	13.04	18.10
Range	2 to 31	8 to 37
IOP at days 2–3 (mmHg)		
Mean	9.58	18.00
Range	2 to 23	7 to 36
IOP at week 1 (mmHg)		
Mean	7.49	19.19
Range	3 to 18	8 to 36
Number of topical antiglaucomatous medications at baseline		
Mean ± SD	3.1 ± 1.3	2.5 ± 1.5
Range	1 to 5	9 to 5

The baseline examination was conducted between 85 and 0 days before surgery with an average of 20.0 ± 26.5 days before surgery, the d1 examination was conducted 1 day after surgery in all patients, the d2–3 examinations has conducted an average of 2.5 ± 0.5 days after surgery, and the w1 examination was conducted an average of 9.1 ± 1.4 days after surgery. Suturolysis was performed in three patients on the first and/or the second day after surgery.

The mean IOP at baseline was 30.04 mmHg (95% CI 26.50 to 33.59) for study eyes and 17.39 mmHg (95% CI 14.49 to 20.30) for fellow eyes. In study eyes with available SD-OCT examination the mean IOP decreased by 17.01 mmHg to a mean of 13.04 mmHg on day 1 (95% CI 8.42 to 17.65, *p* < 0.001, *n* = 18), by 20.46 mmHg to a mean of 9.58 mmHg on days 2 to 3 (95% CI 5.04 to 14.11, *p* < 0.001, *n* = 19), and by 22.55 mmHg to a mean of 7.50 mmHg in week 1 (95% CI 2.45 to 12.52, *p* < 0.001, *n* = 9) after surgery. For fellow eyes, there was no significant change in IOP at all three follow-up periods (*p* > 0.331 respectively). A total of 72% of patients showed a reduction in IOP of ≥ 30% already on day one after surgery.

### Dynamics of BMO-based parameters and RNFL thickness of study eyes

After surgery, BMO-MRW increased from baseline compared to follow-up on d1, d2–3, and w1, along with the reduction of the respective mean IOP at the visit. One day after surgery, the mean BMO-MRW changed by + 26.17 µm, *p* = 0.001 (mean IOP reduction by 17.01 mmHg). This increase persisted on days 2–3 with a mean increase of BMO-MRW of + 25.33 µm, *p* = 0.001 (mean IOP reduction by 20.46 mmHg) and by week 1 with a mean BMO-MRW increase of + 33.17 µm, *p* < 0.001 (mean IOP reduction by 22.55 mmHg). Table 2 details changes in BMO-MRW and the statistical significance of all three-time intervals.

**Table 2:** Mixed regression analysis to compare change in intraocular pressure, Bruch’s membrane minimum rim width and retinal nerve fiber layer thickness before and after surgery

		Study eyes			Fellow eyes		
		Coefficient	95% confidence interval	*p*	Coefficient	95% confidence interval	*p*
IOP (mmHg)	Intercept (Mean at baseline)	30.042	26.497 to 33.587	< 0.001	17.391	14.478 to 20.304	< 0.001
	Day 1	–17.007	–21.62 to -12.395	< 0.001	0.710	–2.578 to 3.999	0.674
	Day 2/3	–20.464	–24.998 to -15.931	< 0.001	0.612	–2.682 to 3.906	0.717
	Week 1	–22.553	–27.589 to -17.518	< 0.001	1.803	–1.799 to 5.405	0.331
BMO-MRW (µm)	Intercept (Mean at baseline)	156.479	125.408 to 187.549	< 0.001	222.465	179.803 to 265.127	< 0.001
	Day 1	26.168	11.937 to 40.398	0.001	–10.403	–20.671 to –0.136	0.054
	Day 2/3	25.325	11.618 to 39.033	0.001	–3.659	–13.478 to 6.16	0.469
	Week 1	33.165	17.477 to 48.852	< 0.001	1.695	–9.159 to 12.55	0.761
RNFL thickness (µm)	Intercept (Mean at baseline)	57.570	51.15 to 63.99	< 0.001	75.783	67.754 to 83.811	< 0.001
	Day 1	2.259	–0.388 to 4.906	0.101	–1.340	–2.449 to –0.23	0.023
	Day 2/3	2.368	–0.207 to 4.944	0.078	–1.384	–2.469 to –0.299	0.016
	Week 1	3.936	0.892 to 6.981	0.015	–0.081	–1.319 to 1.157	0.898
BMO area (mm^2^)	Intercept (Mean at baseline)	2152.917	1950.205 to 2355.628	< 0.001	2107.913	1897.8 to 2318.026	< 0.001
	Day 1	–15.647	–70.461 to 39.168	0.578	–17.868	–50.853 to 15.117	0.294
	Day 2/3	–6.523	–59.317 to 46.27	0.810	–15.665	–47.208 to 15.879	0.336
	Week 1	–32.764	–93.223 to 27.695	0.293	8.614	–26.256 to 43.485	0.631

**Fig. 1:**
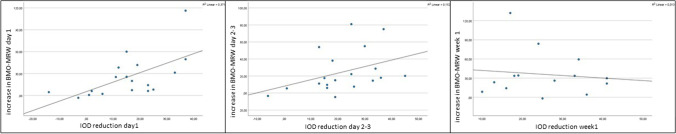
Scatter plots showing a correlation between the increase in BMO-MRW and reduction in IOP on day 1, days 2–3, and week 1 after surgery. The line symbolizes a linear regression

The increase in BMO-MRW correlated significantly with the reduction of IOP on d1 (Spearman’s rho *ρ* = 0.656, *p* = 0.003) and d2–3 (Spearman’s rho *ρ* = 0.479, *p* = 0.038). There was no statistically significant correlation found between the IOP reduction and the increase in BMO-MRW in w1. Details are shown in Fig. 1.

In 72% of all study eyes, a ≥ 30% reduction in IOP was achieved already on day 1 after trabeculectomy. In a subgroup analysis, these patients showed a mean BMO-MRW increase of + 32.4 ± 32.8% on day 1. In eyes with a less than 30% IOP reduction on day 1, the mean change in BMO-MRW was only + 2.3 ± 3.1% at this time.

Peripapillary RNFL thickness showed a significant increase by on average 3.94 µm one week after surgery (*p* = 0.15), while after d1 and d2–3, the change in RNFL was not statistically significant.

**Fig. 2:**
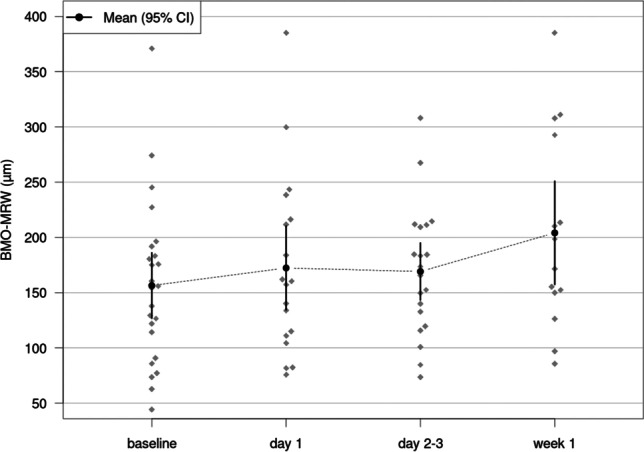
Dynamics of mean BMO-MRW at baseline, on day 1, days 2–3, and at week 1: BMO-MRW significantly increases at all three follow up intervals after surgery compared to baseline before surgery

**Fig. 3:**
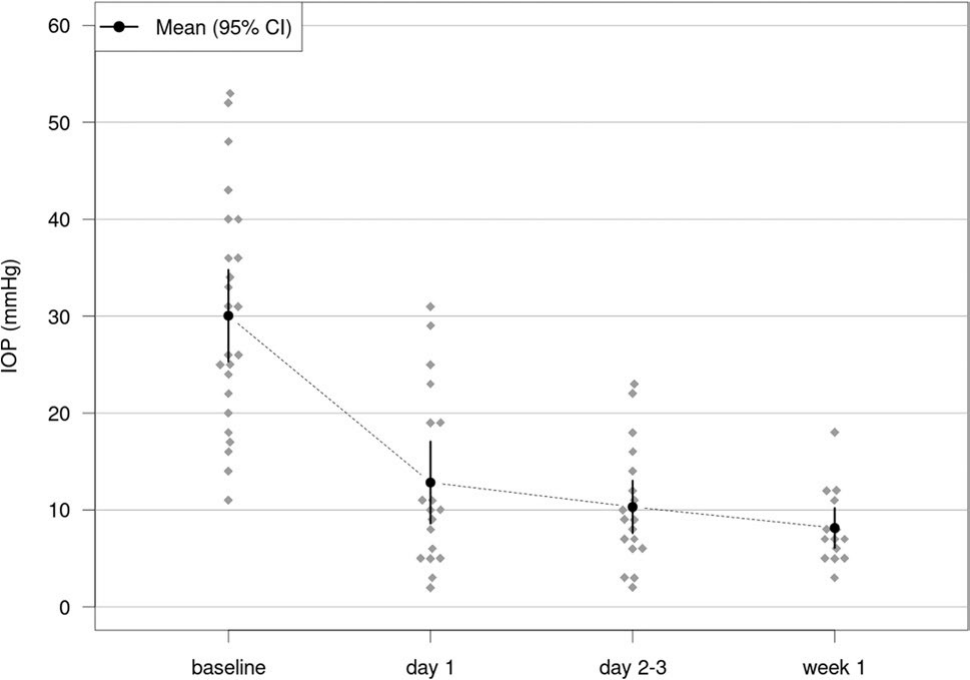
Dynamics of mean intraocular pressure (IOP) at baseline, on day 1, days 2–3, and at week 1

**Fig. 4:**
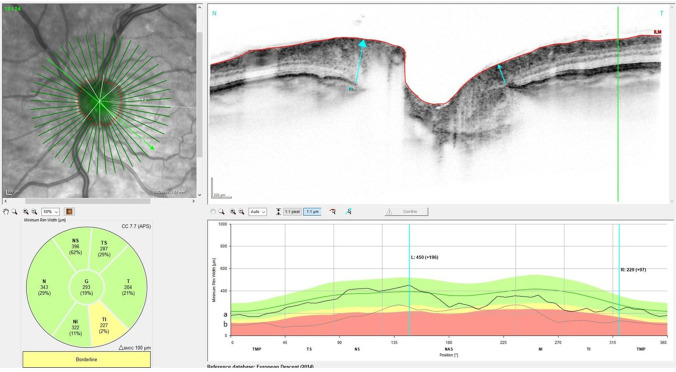
BMO-MRW of an exemplary patient 1 day after trabeculectomy. The black line **a** symbolizes the current BMO-MRW values, while the gray line and **b** symbolizes the baseline BMO-MRW. A significant increase in BMO-MRW can be seen

BMO area showed no significant changes from baseline compared to all follow-up intervals (*p* ≥ 0.29, respectively). Table 3 displays detailed results of BMO-MRW measurements at the different examination time points of the study eyes. Figures 2 and 3 display average BMO-MRW measurements and mean IOP of all study eyes over time. Figure 4 displays the SD-OCT data output of an exemplary patient on day 1 after surgery.

**Table 3:** Global and sectorial Bruch’s membrane minimum rim width (BMO-MRW) in study and fellow eyes

	(µm)	Global	Nasal	Nasal superior	Nasal inferior	Temp	Temp. superior	Temp. inferior
Study eyes	Baseline	156.5 ± 74.2	180.6 ± 73.2	169.9 ± 85.4	191.1 ± 88.3	125.6 ± 67.5	129.6 ± 88.6	150.4 ± 109.3
	Day 1	172.4 ± 82.2	193.0 ± 79.0	193.9 ± 91.6	207.1 ± 101.7	137.4 ± 79.1	158.3 ± 100.4	171.2 ± 117.8
	Day 2/3	169.2 ± 59.0	194.3 ± 65.6	184.8 ± 72.0	201.7 ± 77.3	140.0 ± 48.1	155.0 ± 74.6	151.3 ± 83.0
	Week 1	204.1 ± 89.3	235.9 ± 81.3	226.9 ± 112.8	237.6 ± 102.8	170.2 ± 81.2	188.6 ± 116.3	179.2 ± 130.7
Fellow eyes	Baseline	222.5 ± 105.6	238.9 ± 104.6	249.5 ± 122.6	265.5 ± 111.3	178.8 ± 93.1	211.0 ± 134.0	232.7 ± 153.8
	Day 1	212.7 ± 122.4	217.0 ± 109.5	240.1 ± 142.2	249.0 ± 131.8	187.2 ± 103.9	209.6 ± 165.3	224.4 ± 169.8
	Day 2/3	204.8 ± 83.9	231.4 ± 99.8	216.2 ± 105.3	225.4 ± 102.6	161.3 ± 49.1	185.3 ± 84.0	205.2 ± 106.3
	Week 1	232.4 ± 116.1	245.6 ± 112.0	248.6 ± 135.0	282.6 ± 105.6	196.1 ± 115.6	226.1 ± 142.3	249.1 ± 163.7

### Morphometric SD-OCT parameters in fellow eyes

In the fellow eyes, there was no significant change in BMO-MRW as well as BMO area from baseline compared to all follow-up intervals (*p* ≥ 0.054; *n* ≥ 12, respectively). However, there was a small yet significant decrease in RNFL thickness from 75.78 µm at baseline by 1.34 µm on day 1 (*p* = 0.023, *n* = 16) and by 1.38 µm (*p* = 0.016, *n* = 17) on days 2–3. Table 3 shows details of BMO-MRW measurements at the different examination time points of the fellow eyes.

## Discussion

Understanding potential bias in SD-OCT-based imaging of the ONH’s neuroretinal tissue is crucial for successful and reliable monitoring of glaucoma patients in longitudinal follow-up. The findings of this study can confirm that IOP lowering surgery influences BMO-based morphometric parameters significantly and as early as 1 day after the intervention. The intraday variability of the parameter BMO-MRW was shown to have a mean difference of about 3 μm [17]. Therefore, the increase in BMO-MRW in this study exceeds the intraday variability by around 10 times and seems clinically relevant.

Our findings indicate that there is a small building up of BMO-MRW increase over the first week after surgery while the IOP was continuously lowered during the first week after surgery. (BMO-MRW increased by + 26.17 µm on day 1, by + 25.33 µm on days 2–3 and by + 33.16 µm in week 1; mean IOP at baseline 30.04 mmHg (95% CI 26.50 to 33.59), 13.04 mmHg on day 1, 9.58 mmHg on days 2–3 and 7.50 mmHg in week 1).

The change in BMO-MRW was immediate and dependent on the change of IOP, as there was a significant correlation between the reduction in IOP and the increase in BMO-MRW. This study suggests that the increase in BMO-MRW is caused by the decrease in IOP rather than by the surgery itself. This can be deducted from the fact that those eyes without a reduction in IOP 1 day after surgery also did not show the relevant change in BMO-MRW at this time point.

The IOP on the first postoperative day varied between 2 and 31 mmHg. Therefore the increase in BMO-MRW also varied. No patient in our cohort had clinically relevant choroidal detachment or swelling. Therefore no patients were excluded from the analysis due to choroidal detachment. These complications depend highly on preoperative IOP levels, patients’ age, and other factors. In addition, in our clinical set-up, IOP is assessed using rebound tonometry (Icare) which tends to underestimate the true IOP.

Up to now, the onset, as well as the exact mechanism of structural reversal of disc cupping and increase in BMO-based OCT parameters, has not been fully understood. Development of axonal edema has been one hypothesis; another could be a release of a preoperative compression of the nerve fibers. Up to now, only funduscopically observed changes in the ONH’s cup-to-disc ratio had been described as early as 1 day after an IOP lowering intervention [9]. Two experimental studies in rhesus macaques analyzed the change in OCT-based morphometric parameters within the first hour after trabecular laser-induced IOP-elevation. They found the rim width at the Bruch membrane opening to be affected by an acute increase in IOP also within the first hour after the intervention [18, 19].

In contrast to BMO-based morphometric parameters, peripapillary RNFL thickness seemed not to be influenced by IOP lowering intervention in previous studies. Also, in this study, during the first week after surgery, there was no relevant detectable effect on the peripapillary RNFL thickness.

Therefore, the results of this study are in line with previous studies on changes in morphometric ONH parameters detectable 3 to 12 months after trabeculectomy or other surgical approaches to the treatment of glaucoma. [9, 14, 15]

In this study, we did find a small yet significant decrease in RNFL thickness from baseline compared to day 1 and days 2–3 in fellow eyes by a mean of 1.3 and 1.4 µm respectively without any detectable change in IOP. As Enders et al. found in a previous study, the intraday variability of RNFL thickness had a mean difference of 1.18 μm[17]. In our study, the RNFL thickness of the fellow eyes decreased between baseline and follow-up at days 1 and 2–3 by 1.34 and 1.38 μm, respectively, and therefore in a comparable amount to the intraday variability. However, the decrease in RNFL thickness was statistically significant, indicating a trend of RNFL loss. Given the interval between preoperative baseline and follow-up intervals of 1 day up to 3 months, minor progression of RNFL loss could contribute to the finding.

Since the introduction of OCT for longitudinal follow-up of glaucoma patients, BMO-MRW and -MRA were shown to be more sensitive for early detection of glaucomatous damage [20]. Meanwhile, RNFL thickness was found to have a better longitudinal signal-to-noise ratio than BMO-MRW and -MRA, making RNFL thickness a favorable parameter to monitor progression in glaucoma patients [21]. It remains unclear whether bias by therapeutic interventions was taken into account in these analyses.

In patients with stable IOP, Enders et al. have described comparable intraday variability of BMO-based morphometrics and peripapillary RNFL thickness measured by SD-OCT and no correlation of variability to smaller intraday IOP fluctuations [17].

Nevertheless, BMO-MRW is influenced significantly by larger IOP changes. Acute interventional lowering of IOP during an SD-OCT scan was shown to induce a posterior displacement of the ONH surface and by this create thinning of both BMO-MRW and BMO-MRA [18, 19, 22–24].

RNFL thickness can be biased significantly by scan circle displacement [25], errors in automated segmentation of retinal layers (e.g., by epiretinal gliosis), which occurs disproportionally in eyes with a thinner RNFL thickness, older age, and decreased scan quality [26]. Also, in advanced stages of glaucoma, the parameter RNFL thickness reaches a floor of approximately 40–50 μm and does not decrease any further [27, 28].

It is widely considered best practice in clinical routine to assess a combination of both RNFL thickness as well as BMO-based OCT parameters and functional parameters for monitoring and treating our patients with glaucoma [29]. For BMO-based parameters, potential bias by previous major IOP reduction or elevation must be kept in mind. Changes in BMO-MRW could also occur in case of pronounced IOP fluctuations or a medically induced IOP reduction.

Limitations of our study include a potential selection bias due to exclusion of patients with insufficient postoperative image quality. Most of the examinations excluded from the analysis due to poor image quality were in patients who had anterior chamber bleeding. Those patients often show a delayed IOP reduction after surgery and would probably have shown a delayed SRDC.

Suturolysis was performed in three patients on the first and/or the second day after surgery. Maintaining a lowered IOP is the goal of performing trabeculectomy. When performed, suturolysis aims to lower the IOP after surgery if the target IOP was not reached. In consequence, suturolysis would not cause bias but contribute to the goal of the surgery.

Another limitation is the small sample size of *n* = 24 of this study. Data consistency and prospective enrollment of patients are supportive of our statistically significant findings. Different etiologies of glaucoma have been included in this study. We already know that corneal and scleral biomechanics vary between types of glaucoma. SRDC could also differ between different glaucoma subtypes.

The baseline examination was conducted between 80 and 0 days before surgery with an average of 20.0 ± 26.5 days before surgery, and no additional SD-OCT examination was conducted on the day before the surgery. This could potentially weaken the measured effect of SRDC as the BMO-MRW could have decreased further between the baseline examination and the surgery due to the progression of glaucoma. However, we think this effect should be of limited relevance given the short time between baseline and surgery.

In summary, our study showed structural reversal of disc cupping in BMO-MRW to occur as early as 1 day after IOP lowering intervention and to persist until week 1 with a small building up over the first week after surgery. The increase in BMO-MRW correlated significantly with the reduction in IOP. Ophthalmologists need to consider these changes in BMO-based parameters when evaluating patients’ longitudinal follow-up not only after glaucoma surgery but also after other IOP lowering or raising events.

## Data Availability

Not applicable.
